# *Aedes aegypti* anti-salivary proteins IgG levels in a cohort of DENV-like symptoms subjects from a dengue-endemic region in Colombia

**DOI:** 10.3389/fepid.2022.1002857

**Published:** 2022-11-10

**Authors:** Olayinka M. Olajiga, Alejandro Marin-Lopez, Jenny C. Cardenas, Lady Y. Gutierrez-Silva, Maria U. Gonzales-Pabon, L. Paulina Maldonado-Ruiz, Matt Worges, Erol Fikrig, Yoonseong Park, Berlin Londono-Renteria

**Affiliations:** ^1^Department of Entomology, Kansas State University, Manhattan, KS, United States; ^2^Department of Internal Medicine, Yale University School of Medicine, New Haven, CT, United States; ^3^Laboratorio Clínico, Hospital Local Los Patios, Los Patios, Colombia; ^4^Laboratorio Clinico, E.S.E Hospital Emiro Quintero Cañizares, Ocaña, Colombia; ^5^Laboratorio Clínico, Hospital Erasmo Meoz de Cúcuta, Cúcuta, Colombia; ^6^Department of Tropical Medicine, School of Public Health and Tropical Medicine, Tulane University of New Orleans, New Orleans, LA, United States

**Keywords:** *Ae. aegypti*, dengue, vector borne disease, febrile, pathogenesis, salivary proteins, immunoglobulin G

## Abstract

Dengue fever, caused by the dengue virus (DENV), is currently a threat to about half of the world's population. DENV is mainly transmitted to the vertebrate host through the bite of a female *Aedes* mosquito while taking a blood meal. During this process, salivary proteins are introduced into the host skin and blood to facilitate blood acquisition. These salivary proteins modulate both local (skin) and systemic immune responses. Several salivary proteins have been identified as immunogenic inducing the production of antibodies with some of those proteins also displaying immunomodulatory properties enhancing arboviral infections. IgG antibody responses against salivary gland extracts of a diverse number of mosquitoes, as well as antibody responses against the *Ae. aegypti* peptide, Nterm-34 kDa, have been suggested as biomarkers of human exposure to mosquito bites while antibodies against AgBR1 and NeSt1 proteins have been investigated for their potential protective effect against Zika virus (ZIKV) and West Nile virus infections. Thus, we were interested in evaluating whether IgG antibodies against AgBR1, NeSt1, Nterm-34 kDa peptide, and SGE were associated with DENV infections and clinical characteristics. For this, we tested samples from volunteers living in a dengue fever endemic area in Colombia in 2019 for the presence of IgG antibodies against those salivary proteins and peptides using an ELISA test. Results from this pilot study suggest an involvement of antibody responses against salivary proteins in dengue disease progression.

## Introduction

Interactions between people, mosquito vectors, pathogens, and environmental factors drive arbovirus transmission (1). Viruses from infected humans may be ingested by a female mosquito during a blood meal, replicate within the mosquito, and spread to tissues including the salivary glands, from where the virus is injected with saliva into a susceptible host during the next blood meal (2). Mosquito saliva not only facilitates blood uptake but also induces the production of antibodies (3). Our previous studies demonstrated that some of these proteins elicit a strong antibody response that is related to the intensity of mosquito bite exposure (4, 5). Research also showed that antibodies against *Aedes* salivary proteins are associated with disease risk (4) and some play a role in dengue virus (DENV) infection (5–9). One example is the 34 kDa protein, observed to decrease type I interferon and anti-microbial peptides, promoting virus replication in human keratinocytes during DENV infection (10) and a significant peptide Nterm-34 kDa has been identified as a highly immunogenic peptide in vertebrate hosts with the IgG antibody level correlating with the intensity of exposure to mosquito bites IgG antibodies (11). Antibodies against *Ae. aegypti* salivary gland extract (SGE) was also found as a biomarker of exposure to mosquito bites (4, 5). Other proteins such as the Neutrophil-stimulating protein 1 (NeSt1), and the AgBR1 have been recently identified to modulate ZIKV infection and passive immunization with these proteins reduces ZIKV in mice preventing early viral replication and improving the survival rates (12–15) while the role of these salivary proteins in DENV infection is unclear.

DENV is a single-stranded positive RNA virus belonging to the Flaviviridae family with four serotypes (DENV 1–4) causing an acute febrile illness in humans (16). It is transmitted mainly by both *Aedes aegypti* and *Aedes albopictus* mosquitoes. Based on symptoms and disease management, the World Health Organization (WHO) has classified dengue fever severity into three categories in 2019: dengue without warning signs (DWOWS), dengue with warning signs (DWWS), and severe dengue (SD) (17). Although 70% of DENV infections are asymptomatic, headache, fever, rash, and vomiting are common symptoms that can last up to a week (18) but between 0.5 and 5% of dengue infections progress to the severe stage (17).

The global prevalence of DENV has increased significantly in recent decades, with an estimated 100–400 million infections per year putting roughly half of the world's population at risk (19). DENV has emerged as a major public health issue in the tropics and subtropics, with serious social and economic consequences (20). In Latin America, Colombia is one of the countries with the highest dengue fever incidence rates, with the four dengue serotypes circulating concurrently at any given year (21, 22). The department of Norte de Santander was among Colombia's departments with the highest dengue fever reported cases during the most recent dengue fever epidemic in 2019, which resulted in a total of 127,553 cases in Colombia (23).

In this study, we investigated the potential relationship between the levels of IgG antibodies against SGE, Nterm-34 kDa peptide, AgBR1, and NeSt1 and the clinical presentations of dengue fever in volunteers living in a DENV endemic area in Colombia during the 2019 outbreak. This is the first study to evaluate a relationship between the two recently discovered salivary proteins AgBR1 and NeSt1 and dengue fever.

## Materials and methods

### Ethical considerations

The protocols and methods for this study were reviewed and approved by the Kansas State University Ethics Review Board (IRB#8952, approval date- 10/11/2017). The Cúcuta and Ocaña Hospital Board also approved the methods and authorized the implementation of the study in their institutions. Before sample collection, each potential participant (adults, guardians, or parents of minors) was given a thorough explanation of the study's objectives, and written informed consent was obtained from individuals willing to participate. Blood samples were collected in compliance with the regulations on ethics of research in human participants for Colombia and the United States.

### Geographical location of sampled participants

This study was conducted in the department of Norte de Santander of Colombia. The department has 6 regions with 40 municipalities. Norte de Santander's capital, Cúcuta is located in the eastern part of the department with a level 3 hospital (advanced), where patients with severe diseases are transferred from level 1 and 2 facilities (basic and intermediate, respectively), while the second-largest city—Ocaña located in the western part with fewer densely populated areas, has a level 2 hospital (intermediate) where patients with mild diseases can seek health care. Norte de Santander shares a border with Venezuela and is the primary trade route between the two countries. Agriculture is the most important economic activity in the Norte de Santander Department. DENV infections peak in this region between mid-August and mid-October, as well as between December and February, with 1,100 mm of annual rainfall between rainy seasons March–June and September–December (24).

### Sample collection and dengue fever diagnosis

Between January and December 2019, blood samples (5 mL) were collected in dry tubes from all volunteers who reported DENV-like symptoms and within 3–15 days of fever seeking medical care at the Hospital Universitario Erasmo Meoz in Cúcuta and the Hospital of Emiro Quintero Cañizares in Ocaña. Serum was obtained from the whole blood and kept at −20°C until testing. A total of 201 DENV infected patients were enrolled in the study. Participants were categorized into DWOWS, DWWS, and severe dengue according to the WHO dengue fever classifications (17). Also, non-febrile volunteers (*n* = 22) and febrile DENV negative patients (*n* = 39) were included in the study. A questionnaire was used to record participants demographics such as age, gender, place of residence, and the number of people living in each household. To determine DENV infection in serum, an aliquot of each sample was tested DENV positive using a DENV (NS1)-based IgM ELISA, RDT Xerion DENGUE-NS1 antigen (Xerion—IMEX group, Bogota), or qPCR testing following factory recommendations. Once dengue was confirmed, participants were categorized into DWOWS, DWWS, and severe dengue according to the WHO dengue fever classifications (17). A questionnaire was used to record participants demographics including age, gender, place of residence, and the number of people living in each household. No other demographic information was recorded from these patients. It is important to state that these are “convenience samples” collected without prior sample calculations.

### Testing of IgG antibodies against *Ae. aegypti* salivary gland and proteins

The levels of human IgG antibodies against mosquito salivary proteins were measured using the ELISA protocol. We used whole salivary proteins from salivary glands dissected from *Ae. aegypti* mosquitoes as described in Londono-Renteria (4) and Nterm-34 kDa peptide published by Elanga Ndille et al. (11). Both AgBR1 and NeSt1 proteins were expressed in the Drosophila S2 cell line, as previously described (12, 13). The ELISA procedures were optimized using checkerboard titration. ELISA microtiter plates (Santa Cruz Biotechnology, Dallas, TX) (96-well/per salivary protein antigen) were coated with 50 μl/well of 1 μg/ml SGE, 2 μg/ml Nterm-34 kDa peptide and AgBR1, or 5 ug/ml NeSt1 prepared in the coating solution (Kierkegaard and Perry Laboratories, Gaithersburg, MD) and incubated overnight at 4°C. The plates were washed once with 1X PBS + 0.1 percent Tween 20 (Sigma–Aldrich, St. Louis, MO) wash solution before being blocked for 1 h at 37°C with 2% milk powder in wash solution (blocking buffer). Plates were then incubated at 4°C overnight with a 50 μl/well 1:100 dilution of patient sera in blocking buffer for SGE and Nterm-34 kDa peptide, and a 1:50 dilution of patient sera in blocking buffer AgBR1 and NeSt1 proteins. The next day, plates were washed three times with wash solution before being incubated for 2 h with 50 μl/well of a 1:1,000 dilution of horseradish peroxidase-conjugated goat anti-human IgG (Abcam, AB6858). Colorimetric development of plates was achieved using 50 μl/well tetra-methyl-benzidine (Biolegend; San Diego, CA) incubated at room temperature (37°C) for 2 min (SGE and AgBR1) and 3 min (Nterm-34 kDa and NeSt1). The reaction was stopped with 50 μl/well of 1 M phosphoric acid, and the absorbance at 450 nm was measured.

Antibody levels were expressed as the ΔOD value: Δ*OD* = *ODx* − *ODb*, where ODx represents the mean of individual OD in both antigen wells and ODb the mean of the blank wells. For each peptide tested, positive controls were used for the normalization of plate-to-plate variations. Assay variation of samples (inter and intra assay) tested in the study was below 20% and it was only included in the analysis of serum samples with a coefficient of variation ≤ 20% duplicates between duplicates.

### DENV serotype detection

Serum samples were homogenized in TRI Reagent (Zymo Research, Cat# R2050-1-200) for viral RNA extraction. RT-qPCR was used to determine DENV serotype using primers and probes from the multiplex CDC DENV1–4 real-time RT-qPCR kit (Catalog# KK0128) and the Luna Universal Probe One-Step RT-qPCR Kit (Catalog# E3006S). The following were the RT-qPCR conditions used on the Roche Light Cycler 480: RT Step 1: 55°C for 30 min, then 95°C for 2 min. Forty-five cycles of amplification (95°C for 15 s, 60°C for 1 min), and 4°C for 30 s for cooling down. Positive control of DENV1–4 from C6/36 cell culture supernatant was used. Molecular grade water was used as a negative control instead of RNA during qRT-PCR runs. DENV RNA was detected using a reporter dye amplification curve, DENV1- FAM, DENV2- VIC, DENV3- TEXAS RED, and DENV4- CY5.

### Statistical analysis

The difference between two independent groups (i.e., antibody levels between dengue positive and dengue negative subjects) was determined using the Mann-Whitney test with a *p* < 0.05. A comparison of more than three groups was tested with the Kruskal–Wallis test while the correlation between two independent parameters was done using the Spearman correlation method. To perform the Cox proportional hazards model, AgBR1 IgG, NeSt1 IgG, and Nterm-34 kDa IgG measures were included as continuous variables and as such its interpretation represents a jump from 0 to 1. Reference categories for the categorical covariates included in the Cox PH model are: “male” for sex and “none” for preventive measures. Age and IgG measures are included as continuous covariates. Only two levels are noted for Municipality (Cucuta and Ocana). All statistical analysis was performed using GraphPad Prism, version 9.2.0 (GraphPad Software Inc., La Jolla, CA).

## Results

### Cohort characteristics

A total of 201 DENV-infected patients who visited two different levels of Colombia healthcare facility in 2019 were included in the cohort: 43.2% (87) patients from Cúcuta hospital and 56.7% (114) from Ocaña hospital. DENV IgM testing revealed 100% (87) of Cúcuta patients was IgM+, and 80.7% (92) of Ocaña patients was IgM+. The Ocaña DENV IgM- (4) patients were further tested using DENV-NS1 and was confirmed positive. When it comes to dengue fever, 75.9% (66) of DWOWS patients, 24.1% (21) of DWWS patients visited Cúcuta health facility, while 32.5% (37) of DWOWS patients, 67.5% (77) of DWWS patients visited Ocaña health facility. The cohort's age ranged from 6 months to 67 years, with a median age of 13 years. The patients experienced dengue symptoms ranging from day 1 to 15 days with median day of 6 days (Table 1).

**Table 1:** Cohort characteristics by hospital level.

**Location**	***n***	**Gender**		**Dengue testing**			**Dengue fever classification**		**Age median (range)**	**Symptom days median (range)**
		**M**	**F**	**DENV IgM+**	**NSI IgM+**	**PCR +**	**w/o warnings**	**w/warnings**		
Cúcuta	87	43	44	87	–	–	66	21	14.5 (0–67 years)	6 (1–13 days)
Ocaña	114	48	66	92	4	47	37	77	10.1 (0–53 years)	6 (3–15 days)
Total	201	91	110	179	4	47	103 (51.2%)	98 (48.8%)	13 (0–67 years)	6 (1–15 days)

### Duration of symptoms was associated with anti-salivary proteins IgG level

**Figure 1:**
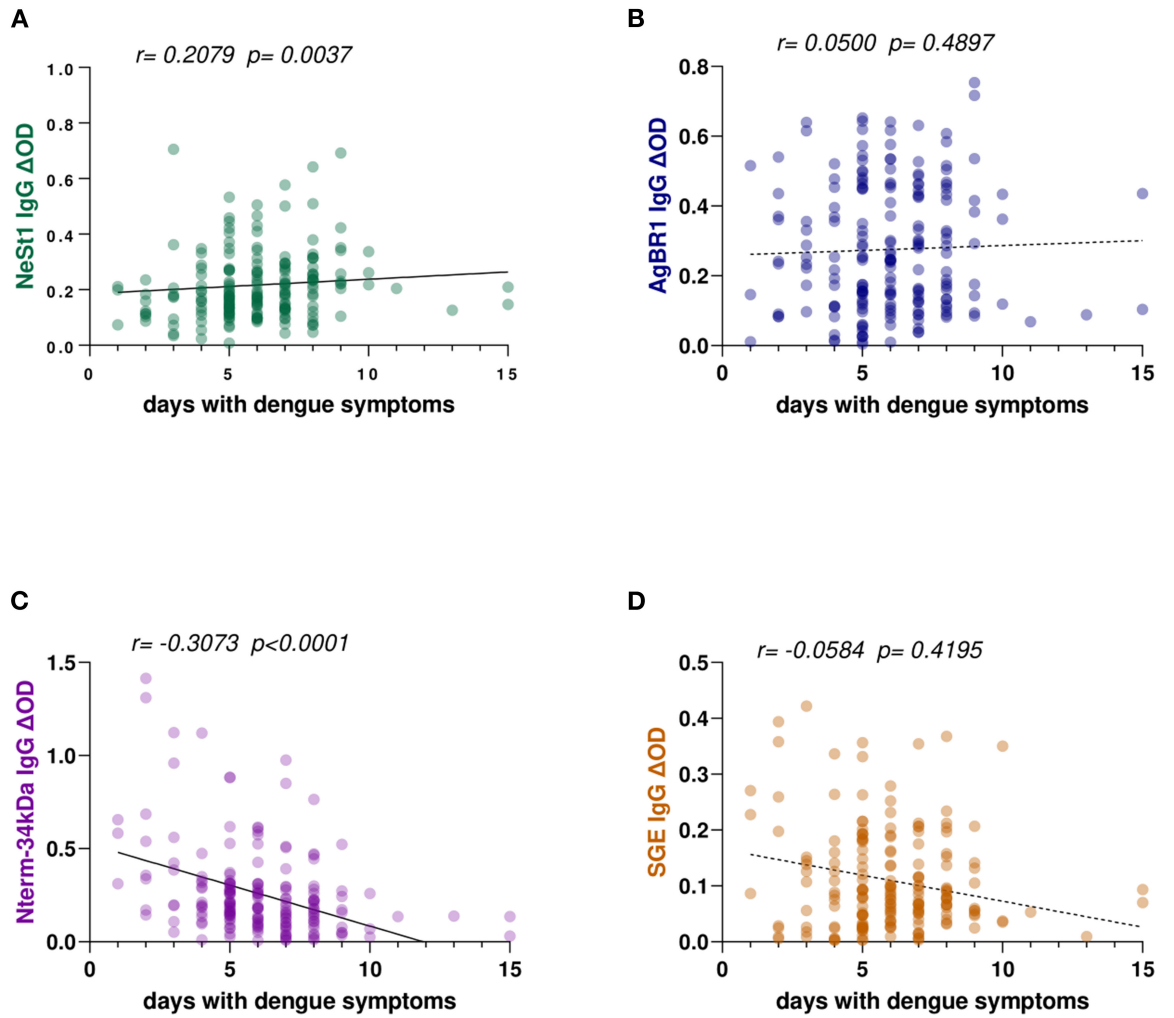
*Ae. aegypti* SGE, Nterm-34 kDa peptide, NeSt1 protein, and AgBR1 protein IgG antibody levels in relation to patients' days with symptoms of dengue fever. **(A)** Green color- NeSt1 protein **(B)** blue color- AgBR1 protein **(C)** purple color- Nterm-34 kDa peptide **(D)** brown color- SGE. Individual IgG levels are represented by the colored dots and a regression line with 95% confidence bands passes through the mean is shown. Antibody levels are measured in units of OD (optical density). The “*r*” and “*p*” values were measured using the pairwise non-parametric Spearman correlation test.

The duration of dengue-like symptoms in our study participants ranged from 1 to 15 days. We ran a correlation analysis between the level of anti-salivary protein IgG antibodies and the number of days from the onset of symptoms; both NeSt1 and Nterm-34 kDa peptide IgG antibodies had a weak significant correlation with days with dengue symptoms. NeSt1 IgG had a weak positive correlation with number of days with dengue symptoms (Spearman's correlation, *r* = 0.2079; *p* = 0.0037; Figure 1A) while Nterm-34 kDa IgG had a weak negative correlation with number of days with dengue symptoms (Spearman's correlation, *r* = −0.3073; *p* < 0.0001; Figure 1C). However, AgBR1 and SGE IgG antibodies had no significant correlation with days with dengue symptoms (Figures 1B,D).

### Anti-salivary proteins IgG levels differ between dengue fever classifications

**Figure 2:**
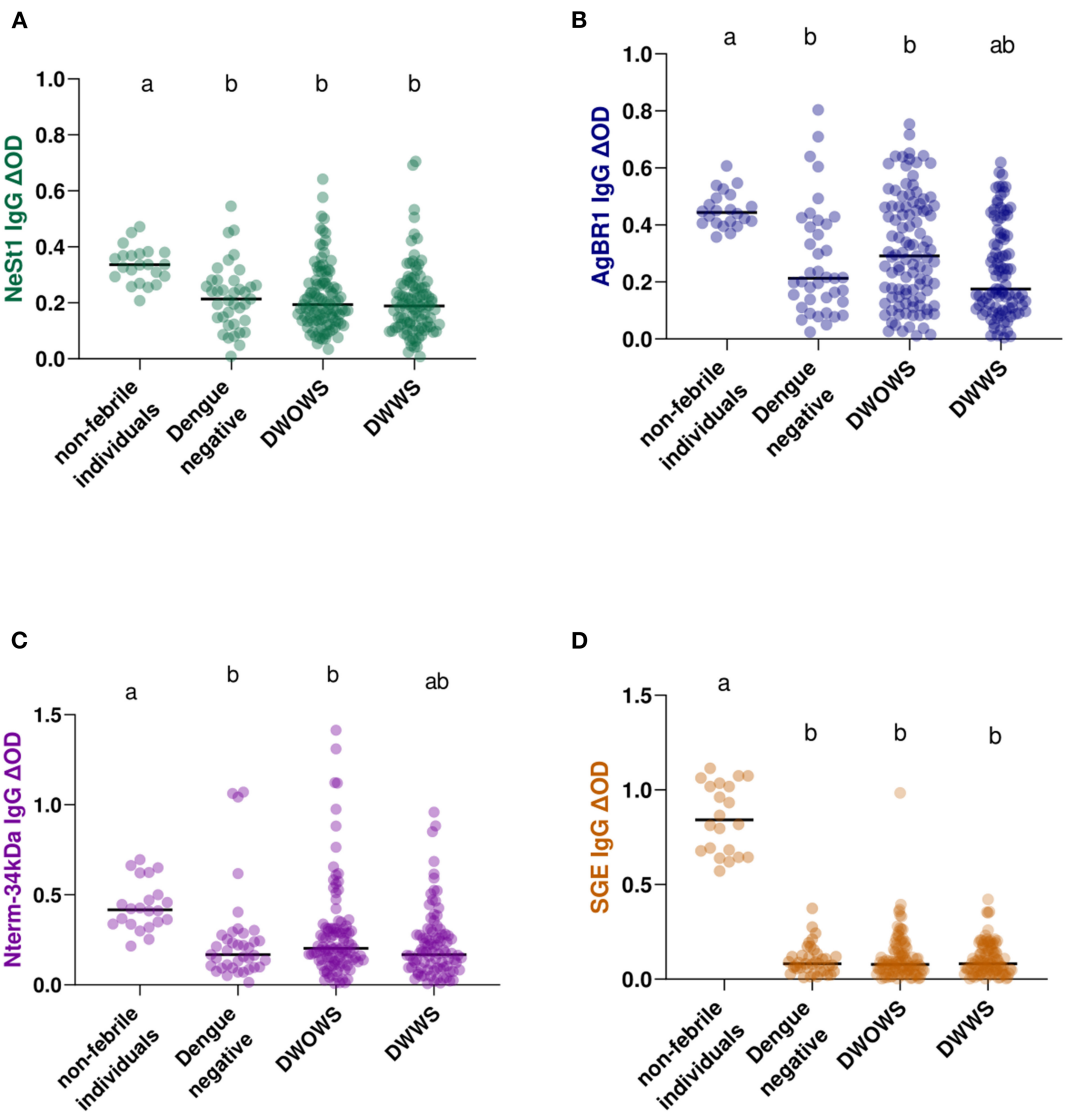
IgG antibody levels of *Ae. aegypti* SGE, Nterm-34 kDa peptide, AgBR1 protein, and NeSt1 protein comparison between 22 non-febrile volunteers, 39 febrile patients with dengue negative result [DENV (NS 1)-based IgM and RDT Xerion DENGUE-NS1 antigen], 103 DWOWS, and 98 DWWS. **(A)** Green color- NeSt1 protein **(B)** blue color- AgBR1 protein **(C)** purple color- Nterm-34 kDa peptide **(D)** brown color- SGE. The individual IgG levels are represented by the colored dots and horizontal red lines represent medians of group individual antibody responses. Antibody levels are measured in units of OD (optical density). Letters indicate statistically significant differences between groups compared with *p* < 0.05 measured using the pairwise non-parametric Mann–Whitney test.

Based on the dengue fever classification comparison of anti-salivary proteins IgG, we observed that non-febrile participants presented a higher level of anti-salivary IgG compared with febrile patients (either dengue positive or negative) in all salivary proteins tested (Figure 2). Interestingly, both AgBR1 and Nterm-34 kDa IgG levels were significantly higher in individuals presenting DWOWS than in those presenting DWWS (Mann-Whitney test, AgBR1 IgG *p* = 0.0053; Nterm-34 kDa IgG *p* = 0.0171; Figures 2B,C) while both levels of NeSt1 IgG and SGE IgG were not significantly different between classifications (Mann-Whitney test, NeSt1 IgG *p* = 0.3533; SGE IgG *p* = 0.7821; Figures 2A,D). Using Cox hazard ratios to evaluate the relevance of the AgBR1 levels association to dengue outcome, we found that for every one unit increase in AgBR1 levels, the hazard of having DWWS decreased by 81%. Similarly, for every one unit increase in NeSt1 levels, the hazard of having signs of DWWS dengue decreased by 88%. The likelihood ratio test is only significant for the AgBR1 and NeSt1 model at an alpha level of 0.05 (Supplementary Table 1).

### Levels of anti-salivary proteins IgG between geographical locations

**Figure 3:**
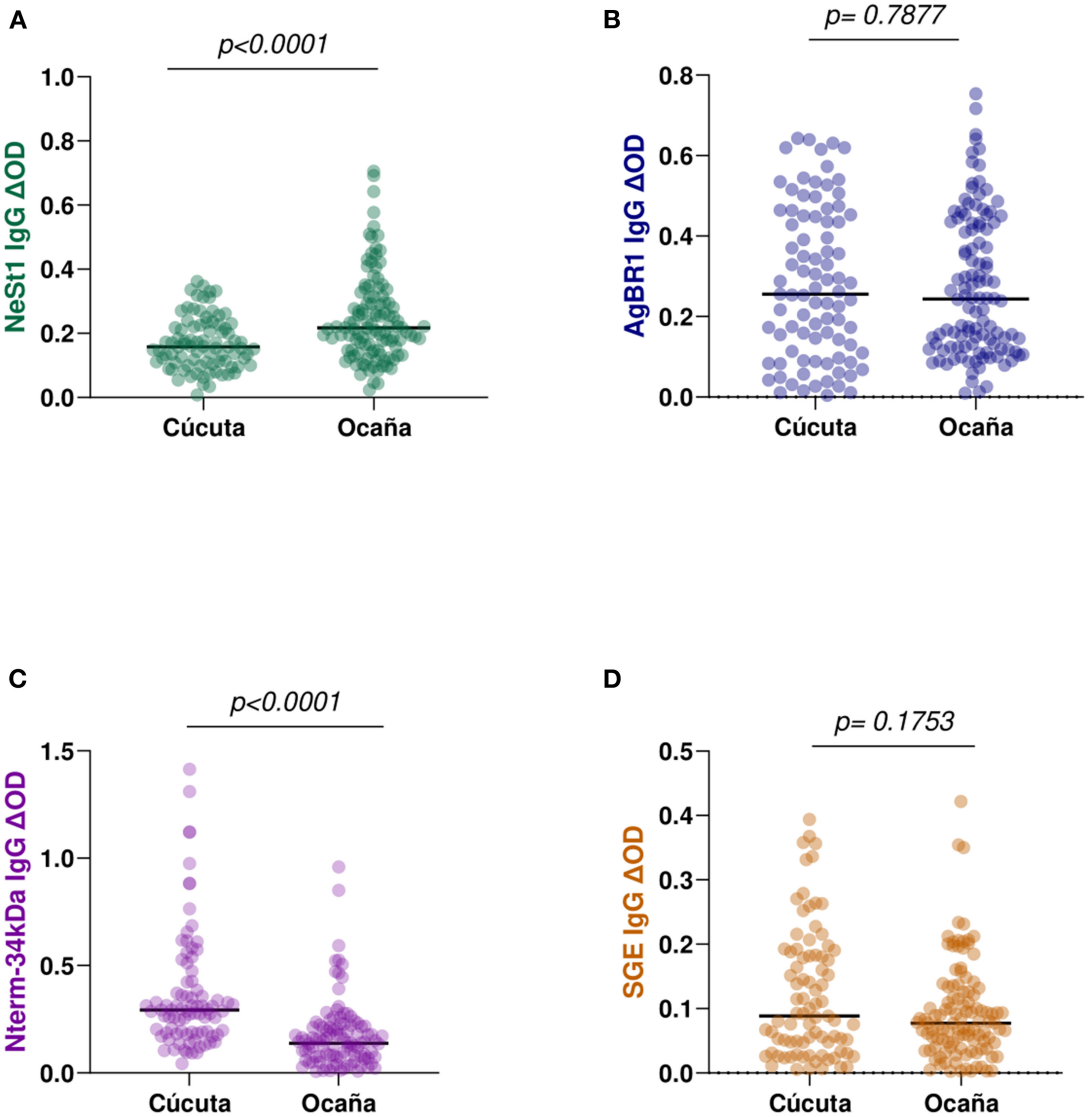
IgG antibody levels against *Ae. aegypti* salivary proteins between two major municipalities of Norte de Santander; Cúcuta (*n* = 87) and Ocaña (*n* = 114). **(A)** Green color- NeSt1 protein **(B)** blue color- AgBR1 protein **(C)** purple color- Nterm-34 kDa peptide **(D)** brown color- SGE IgG antibody levels. Individual IgG levels are represented by colored dots and horizontal red lines represent medians of group individual antibody responses. Antibody levels are measured in units of OD (optical density). “*p* < 0.05” were measured using the pairwise non-parametric Mann–Whitney test.

**Figure 4:**
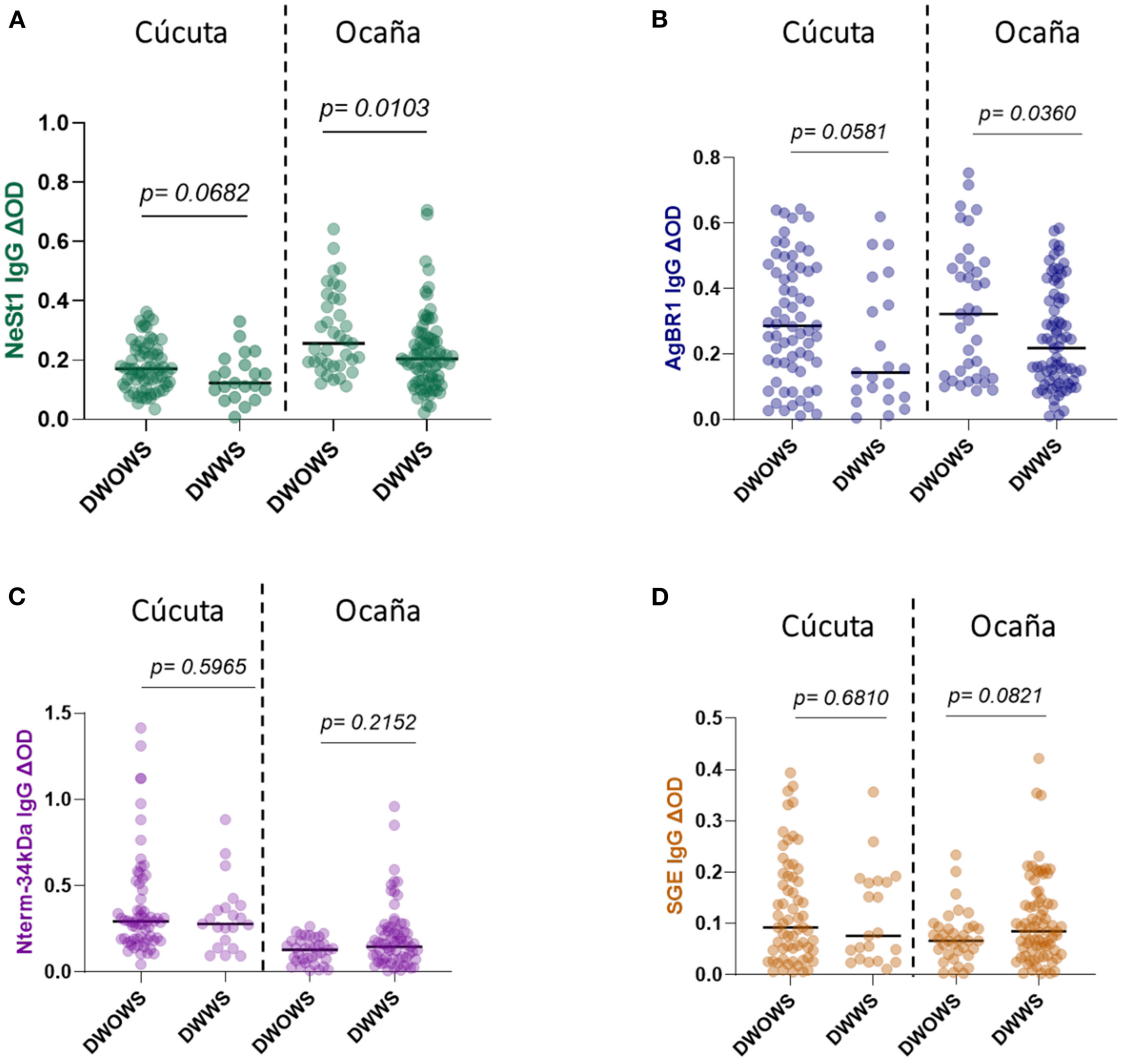
IgG antibody levels of *Ae. aegypti* SGE, Nterm-34 kDa peptide, AgBR1 protein, and NeSt1 protein comparison between DWOWS, and DWWS depending on hospital location **(A)** NeSt 1 protein; left -Cúcuta patients, right- Ocaña patients **(B)** AgBR1 protein; left -Cúcuta patients, right- Ocaña patients **(C)** Nterm-34 kDa peptide; left -Cúcuta patients, right- Ocaña patients **(D)** SGE; left -Cúcuta patients, right- Ocaña patients. The individual IgG levels are represented by the colored dots and horizontal red lines represent medians of group individual antibody responses. Antibody levels are measured in units of OD (optical density). “*p* < 0.05” were measured using the pairwise non-parametric Mann–Whitney test.

We compared patients from two hospital locations with different levels. This is because Cúcuta been the department's capital has a level 3 hospital while Ocaña been smaller has a level 2 hospital and samples were collected from both locations. Eighty-seven Cúcuta hospital patients and 114 Ocaña hospital patients were analyzed, and we observed no significant difference in SGE IgG (Mann–Whitney test, *p* = 0.1753; Figure 3D) or AgBR1 IgG (Mann–Whitney test, *p* = 0.7877; Figure 3B). In the case of NeSt1 IgG Cúcuta hospital patients had lower NeSt1 IgG than Ocaña hospital patients (Mann–Whitney test, *p* < 0.0001; Figure 3A), Contrastingly, Nterm-34 kDa IgG levels was significantly higher in Cúcuta hospital patients than in Ocaña hospital patients (Mann–Whitney test, *p* < 0.0001; Figure 3C). When comparing the level of anti-salivary IgG among dengue fever classification between the two different hospital location, it was surprising to observe that only Ocaña patients had significant difference in the level of AgBR1 (Mann–Whitney test, *p* = 0.0360) and NeSt1 (Mann–Whitney test, *p* = 0.0103) between DWOWS and DWWS while Nterm-34 kDa and SGE IgG was not different in the two locations between DWOWS and DWWS (Figure 4).

### No difference in *Ae. aegypti* salivary proteins IgG levels between DENV serotypes

**Figure 5:**
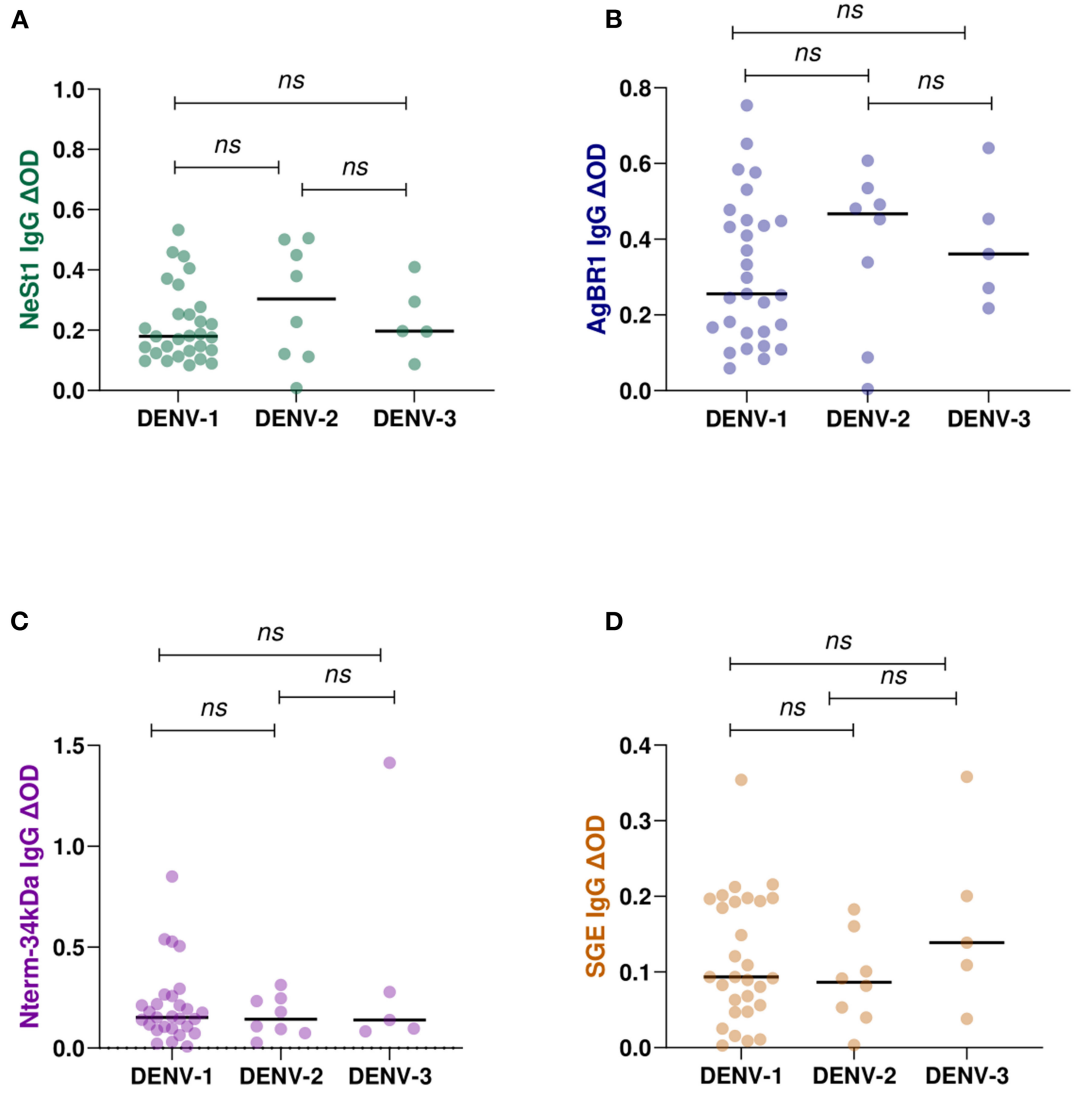
IgG antibody levels against *Ae. aegypti* salivary proteins in qPCR confirmed positive DENY serotypes (DENY 1–3). 29 individuals were DENY-1, 8 individuals were DENV-2, and 5 individuals were DENV-3 confirmed. Individual IgG levels are represented by colored dots and horizontal black lines represent medians of group individual antibody responses. **(A)** green color- NeSt 1 protein **(B)** blue color- AgBR1 protein **(C)** purple color- Nterm-34 kDa peptide **(D)** brown color- SGE. Antibody levels are measured in units of OD (optical density). “*p* < 0.05” were measured using the non-parametric Mann–Whitney test.

The levels of anti-salivary proteins IgG in confirmed dengue serotypes were examined. Of the 42 total identified multiplex qPCR dengue serotypes, DENV-1 (*n* = 29), DENV-2 (*n* = 8), and DENV-3 (*n* = 5) were confirmed, and these were used for the DENV serotype and anti-salivary protein IgG analysis. Pair-wise comparison using the non-parametric Mann-Whitney test revealed no significant differences between each anti-salivary protein IgG level and DENV serotypes (Figure 5).

### Age and gender had no influence on the levels of *Ae. aegypti* salivary proteins IgG in dengue patients

**Figure 6:**
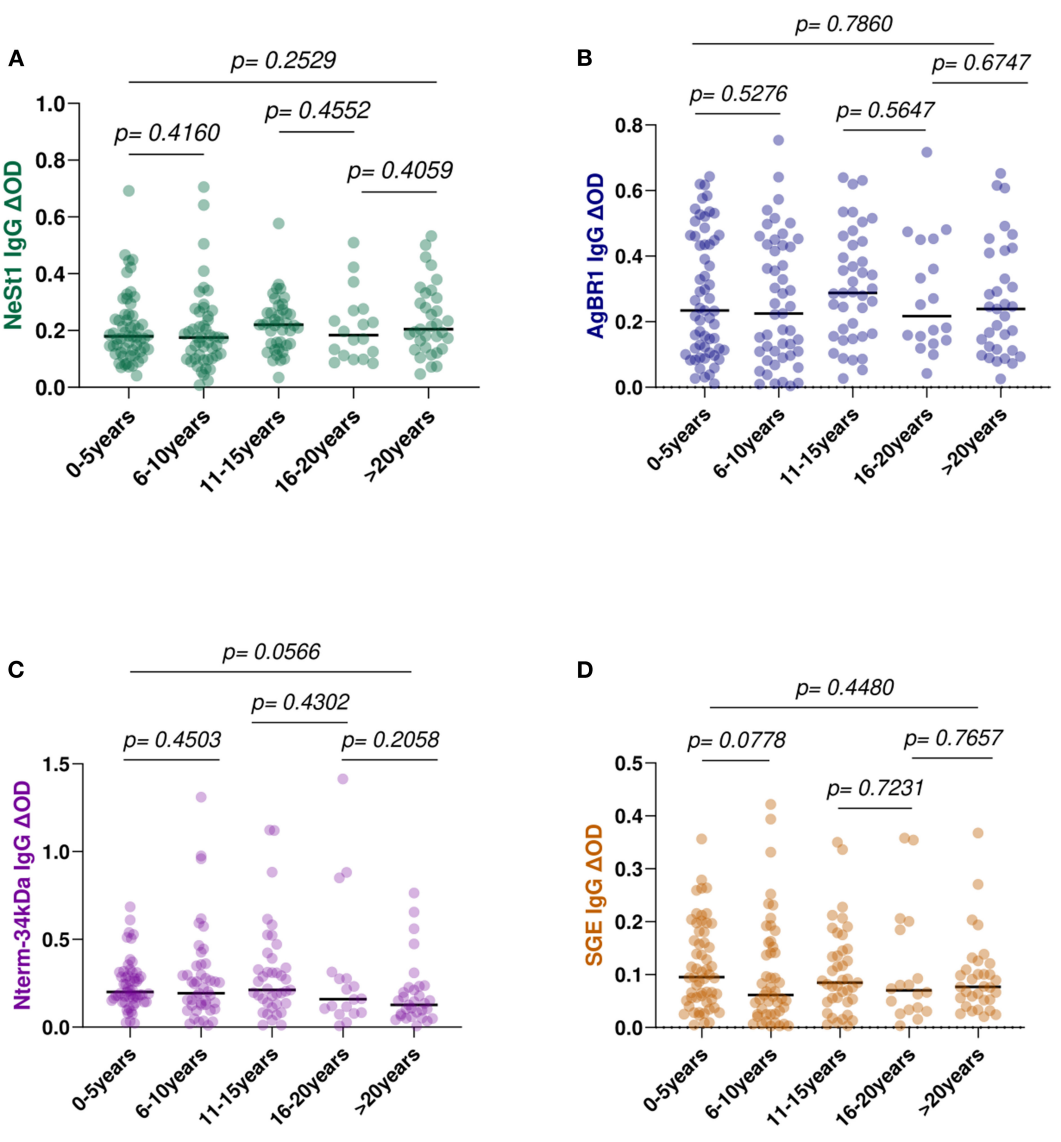
Anti-salivary protein IgG levels comparison between age group classification: 0–5 years, 6–l0, 11–15, 16–20, and above 20 years **(A)** NeSt1 IgG **(B)** AgBR1 IgG **(C)** Nterm-34 kDa IgG **(D)** SGE IgG. The individual IgG levels are represented by the colored dots and horizontal red lines represent medians of group individual antibody responses. Antibody levels are measured in units of OD (optical density). “*p* < 0.05” were measured using the pairwise non-parametric Kruskal–Wallis test for multiple comparisons and the Mann–Whitney testing for comparison between two groups.

To determine if age influences the levels of anti-salivary IgG in dengue patients, we assessed the level of anti-salivary proteins IgG in accordance with the age categories of 0–5, 6–10, 11–15, 16–20, and above 20 years. Fifty-nine patients are within the age range of 0–5 years, 49 patients within the age range of 6–10 years, 41 patients within the age range of 11–15 years, 18 patients are within the age range of 16–20 years, and 33 patients are above 20 years of age. Among the various age groups tested using the Kruskal–Wallis test, NeSt1 IgG had a *p* = 0.2529, AgBR1 had a *p* = 0.7860, Nterm-34 kDa had a *p* = 0.0566, and SGE had a *p* = 0.4480 (Figure 6).

**Figure 7:**
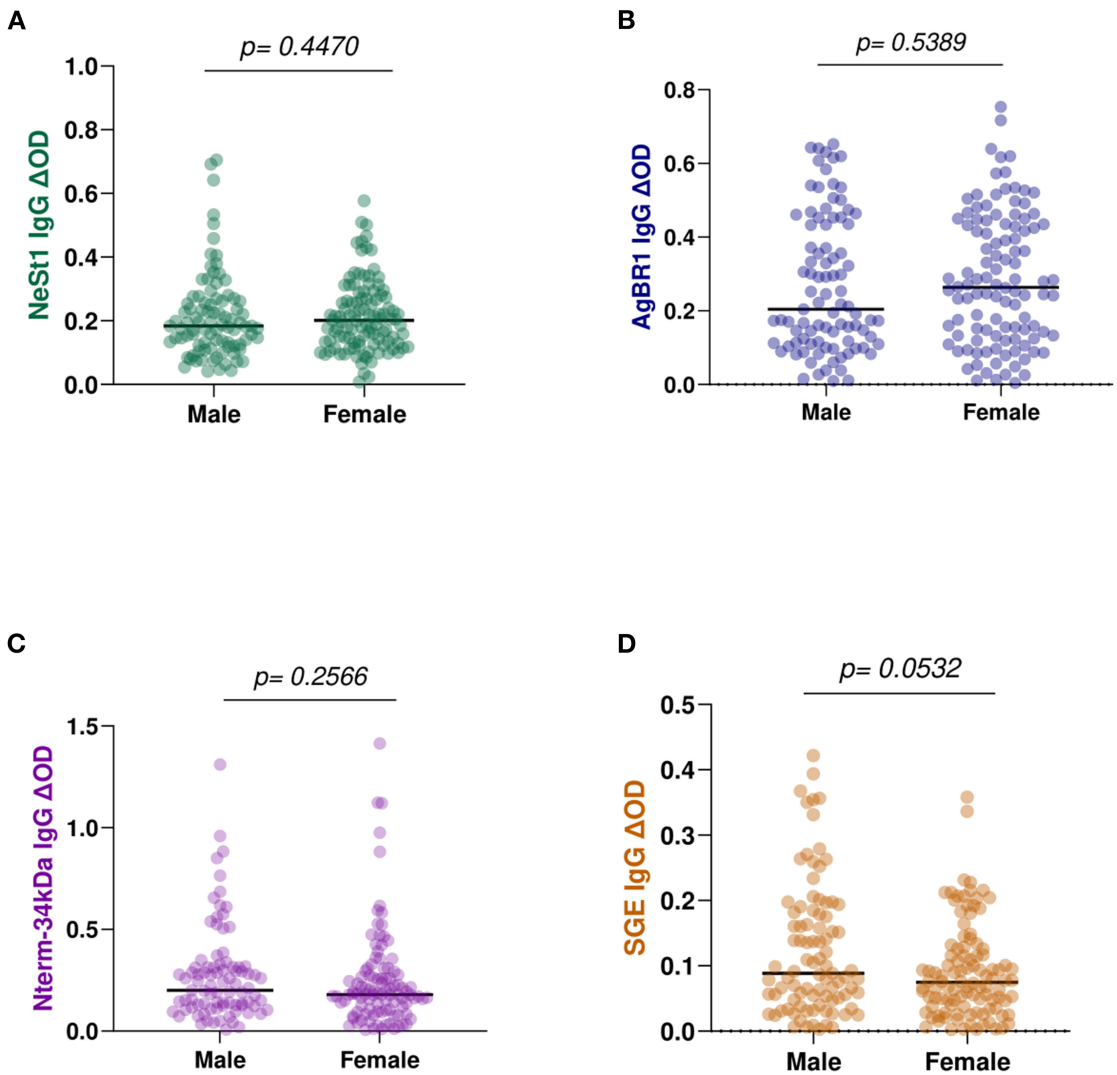
Anti-salivary protein IgG levels comparison between gender: **(A)** NeSt1 IgG **(B)** AgBR1 IgG **(C)** Nterm-34 kDa IgG **(D)** SGE IgG. The individual IgG levels are represented by the colored dots and horizontal red lines represent medians of group individual antibody responses. Antibody levels are measured in units of OD (optical density). “*p* < 0.05” were measured using the pairwise non-parametric Mann–Whitney test.

We also evaluated whether there were differences associated with gender on the levels of anti-salivary IgG in dengue patients. However, there were no significant differences in the levels of anti-salivary IgG between the 91 men and the 110 females. NeSt1 had a *p* = 0.4470, AgBR1 had a *p* = 0.5389, Nterm-34 kDa had a *p* = 0.2566 and SGE had a *p* = 0.0532 (Mann–Whitney test) (Figure 7).

## Discussion

In addition to targeting arboviruses, host immunity is also directed against mosquito salivary proteins that may be modulating dengue pathogenesis. While the identification of specific mosquito salivary proteins opened the way for the development of serological toolboxes that could allow for the evaluation of human-vector interaction and disease transmission by the same vector (25, 26), it could also be useful for epidemiological investigations, as well as for estimating the risk of transmission and the effectiveness of vector control measures. Antibodies against *Ae. aegypti* salivary gland extract have been proven to be useful to evaluate seasonal exposure to mosquito bites (27). These anti-mosquito SGE antibodies are also short-lived and indicative of recent mosquito contact and wane when exposure to specific mosquito species is not sustained (4, 28–30). Our studies also suggest that location influences the level of SGE IgG with residents of houses harboring aquatic stages of *Ae. aegypti* presenting higher *Ae. aegypti* SGE IgG than people living in houses with no mosquitoes breeding (31). We also reported that febrile viremic individuals presented higher antibody levels against whole SGE than febrile non-viremic individuals (4).

In this study, we observed that non-febrile volunteers had higher IgG antibodies than dengue patients. These findings could imply that people may be exposed to more non-infected bites early in the transmission season and may have developed a stronger immune response against salivary proteins, which could act as a protective mechanism against DENV infection. This hypothesis has yet to be proven and will require additional research to confirm. However, a recent study by Manning et. al., showed that high levels of seropositivity to *Ae. aegypti* salivary proteins are associated with future development of dengue infection (32). In this study, we observed that Nterm-34 kDa peptide and AgBR1 IgG antibodies distinguish between dengue disease progression, i.e., dengue without warnings had higher IgG levels than dengue with warnings, indicating that the higher the antibodies, the less severe the symptoms/infection. Although the hazard ratio model was only significant for AgBR1, the results suggest that antibodies against salivary proteins may play a role in dengue clinical presentation. Nonetheless, more research is needed to characterize the role of anti-saliva antibodies in disease progression.

Another helpful biomarker for exposure to *Aedes* mosquito bites has been the IgG response to the Nterm-34 kDa salivary peptide (33), although no conclusive connection to dengue risk has been shown. Symptoms of dengue can range from asymptomatic or mild to severe symptoms that can last up to 15 days (34). Diverse clinical manifestations of dengue prompted the WHO to classify dengue symptoms into three categories of severity; DWOWS, DWWS, and severe dengue (17). Assessing the levels of anti-salivary proteins IgG among dengue fever classification revealed that dengue DWOWS had higher AgBR1 and Nterm-34 kDa peptide IgG levels than those DWWS suggesting that the higher the antibody levels, the less severe the symptoms/infection. However, when location was used to group patients, Nterm-34 kDa peptide IgG level was not significantly different between the dengue fever classes which suggested that Nterm-34 kDa peptide IgG might be a reliable biomarker to detect variation in human exposure to *Ae. aegypti* bite (11) but cannot differentiate between dengue severity. Nevertheless, antibodies against AgBR1 were significantly different between dengue severity classification and hospital location which suggests that AgBR1 IgG may be a diagnostic tool to evaluate the risk of dengue fever severity in endemic regions. This could also imply that location abundance of mosquito or exposure to mosquito bites influences immune response to anti-salivary protein as earlier suggested (3, 28). Furthermore, since the AgBR1 antibodies have been shown to delay deadly West Nile virus infection and modulate early Zika virus infection in mice (13, 35), higher level of anti-AgBR1 in DWOWS patients might also suggest its modulating effect on dengue severity at the early stage of disease. However, since we used convenience samples, it is difficult for us to currently determine how exposure to non-infected bites may have strengthened their immune response to salivary proteins and the potential protective mechanism against DENV infection or developing dengue symptoms. IgG against AgBR1 and Nterm-34 kDa involvement in dengue severity is suggested in this pilot, therefore, more research is needed to assess the risk of disease progression to severity through antibodies against these salivary proteins.

During outbreaks, the increased number of dengue cases in the Norte de Santander region is thought to be due to highly favorable climatic conditions for Aedes mosquito breeding in these years, as well as migration from neighboring countries (4). The disease management in this region depends on the severity of the signs and symptoms, with three tiers of hospitals: level 1 hospital (basic) is where patients with mild disease seek medical attention. Level 2 hospital (intermediate) is where patients with mild and moderate disease seek medical attention. Level 3 hospital (advanced) is where severe patients are referred from level 1 or level 2 health care facilities. The Cúcuta hospital is a level 3 health care facility while Ocaña hospital is a level 2 health care facility. When comparing the levels of anti-salivary proteins among patients visiting the two levels of hospital, we observe Cúcuta hospital residents having significantly higher Nterm-34 kDa IgG than Ocaña hospital patients whereas NeSt1 IgG was significantly higher in Ocaña hospital patients than Cúcuta hospital residents. It was surprising that when we grouped the hospital location based on dengue severity, Ocaña patients presented distinct level of NeSt1 and SGE IgG between DWOWS and DWWS while Nterm-34 kDa presented no significant difference in both Cúcuta and Ocaña patients between DWOWS and DWWS. The reasons for these variations are still unknown, but we are currently organizing a prospectus-controlled study to follow patients before and after mosquito exposure in each one of these localities.

Interestingly, in this study the level of anti-salivary proteins did not differ among DENV serotypes suggesting that immune response to salivary protein during dengue infection may not be DENV serotype dependent. Unfortunately, the low sample size of multiplex confirmed dengue serotypes did not allow for more powerful analysis such as correlating monotypic vs. multitypic status of the cohort (32). Surprisingly, we did not find differences in the level of IgG anti-salivary protein among age groups neither was an association of age with the levels of the anti-salivary protein IgG. Previous studies have described a negative correlation between age and level of IgG antibodies mainly because of the nature of these antigens (36, 37). Salivary proteins are known to elicit the formation of IgG4 antibodies associated with the induction of tolerance (38, 39). However, another study found no correlation with age (40). It is possible that local and environmental factors other than those discussed in this study may influence the development of total IgG or specific IgG4.

In summary, the connection between anti-salivary proteins IgG and dengue severity, number of days with dengue symptoms and hospital disease management suggested that AgBR1 and NeSt1 IgG may be valuable tools to assess the risk of dengue severity progression and distinguish between exposure to DENV. It is noteworthy to highlight that AgBR1, regardless of location in Colombia, appears to be the best predictor of dengue severity distinguishing between DWOWS and DWWS.

## Data availability statement

The raw data supporting the conclusions of this article will be made available by the authors, without undue reservation.

## Ethics statement

The studies involving human participants were reviewed and approved by Kansas State University Ethics Review Board (IRB#8952, approval date- 10/11/2017). Written informed consent to participate in this study was provided by the participants' legal guardian/next of kin.

## Author contributions

JC, LG-S, MG-P, and BL-R: sample collection. OO and BL-R: methodology and funding acquisition. OO, BL-R, and MW: data analysis. OO: writing—original draft preparation. OO, AM-L, LM-R, EF, YP, and BL-R: writing—review and editing. OO, YP, and BL-R: visualization. YP and BL-R: supervision. BL-R: project administration. All authors contributed in conceptualization, read, and agreed to the published version of the manuscript.

## Funding

This research was funded by the 2021–2022 Donald C. Warren Scholarship and COBRE-NIH 5P20GM103638-08, BL-R.

## Conflict of interest

The authors declare that the research was conducted in the absence of any commercial or financial relationships that could be construed as a potential conflict of interest.

## Publisher's note

All claims expressed in this article are solely those of the authors and do not necessarily represent those of their affiliated organizations, or those of the publisher, the editors and the reviewers. Any product that may be evaluated in this article, or claim that may be made by its manufacturer, is not guaranteed or endorsed by the publisher.
