# Supplementary material for: Aedes aegypti anti-salivary proteins IgG levels in a cohort of DENV-like symptoms subjects from a dengue-endemic region in Colombia
Source: Front Epidemiol. 2022 Nov 10;2:1002857. doi: 10.3389/fepid.2022.1002857 (PMC10910902; doi:10.3389/fepid.2022.1002857)

Supplementary Table 1

|  | **AgBR1_IgG**  **(LRT p-value=0.03)** | | **NeSt1_IgG**  **(LRT p-value=0.08)** | | **Nterm34kDa_IgG**  **(LRT p-value=0.3)** | |
| --- | --- | --- | --- | --- | --- | --- |
|  | **HR [95% CI]** | **p-value** | **HR [95% CI]** | **p-value** | **HR [95% CI]** | **p-value** |
| Age (years) | 0.98 [0.97, 1.00] | 0.1300 | 0.99 [0.97, 1.01] | 0.1600 | 0.99 [0.97, 1.01] | 0.2000 |
| Sex (ref cat: male) |  |  |  |  |  |  |
| Female | 1.02 [0.67, 1.55] | 0.9300 | 1.00 [0.66, 1.53] | 0.9900 | 1.01 [0.66, 1.54] | 0.9600 |
| Preventive measure (ref cat: no method) |  |  |  |  |  |  |
| Mosquito net | 1.03 [0.54, 1.94] | 0.9300 | 1.04 [0.55, 1.97] | 0.9000 | 0.88 [0.47, 1.65] | 0.6800 |
| Fumigation | 1.80 [0.74, 4.39] | 0.1900 | 1.82 [0.74, 4.47] | 0.1900 | 1.63 [0.67, 3.94] | 0.2800 |
| Repellents | 0.87 [0.51, 1.50] | 0.6300 | 0.97 [0.57, 1.66] | 0.9200 | 1.07 [0.63, 1.83] | 0.8000 |
| IgG | 0.19 [0.06, 0.65] | ***0.0083*** | 0.12 [0.02, 0.83] | ***0.0320*** | 2.06 [0.67, 6.30] | 0.2100 |

*HR* hazard ratio; *CI* confidence interval


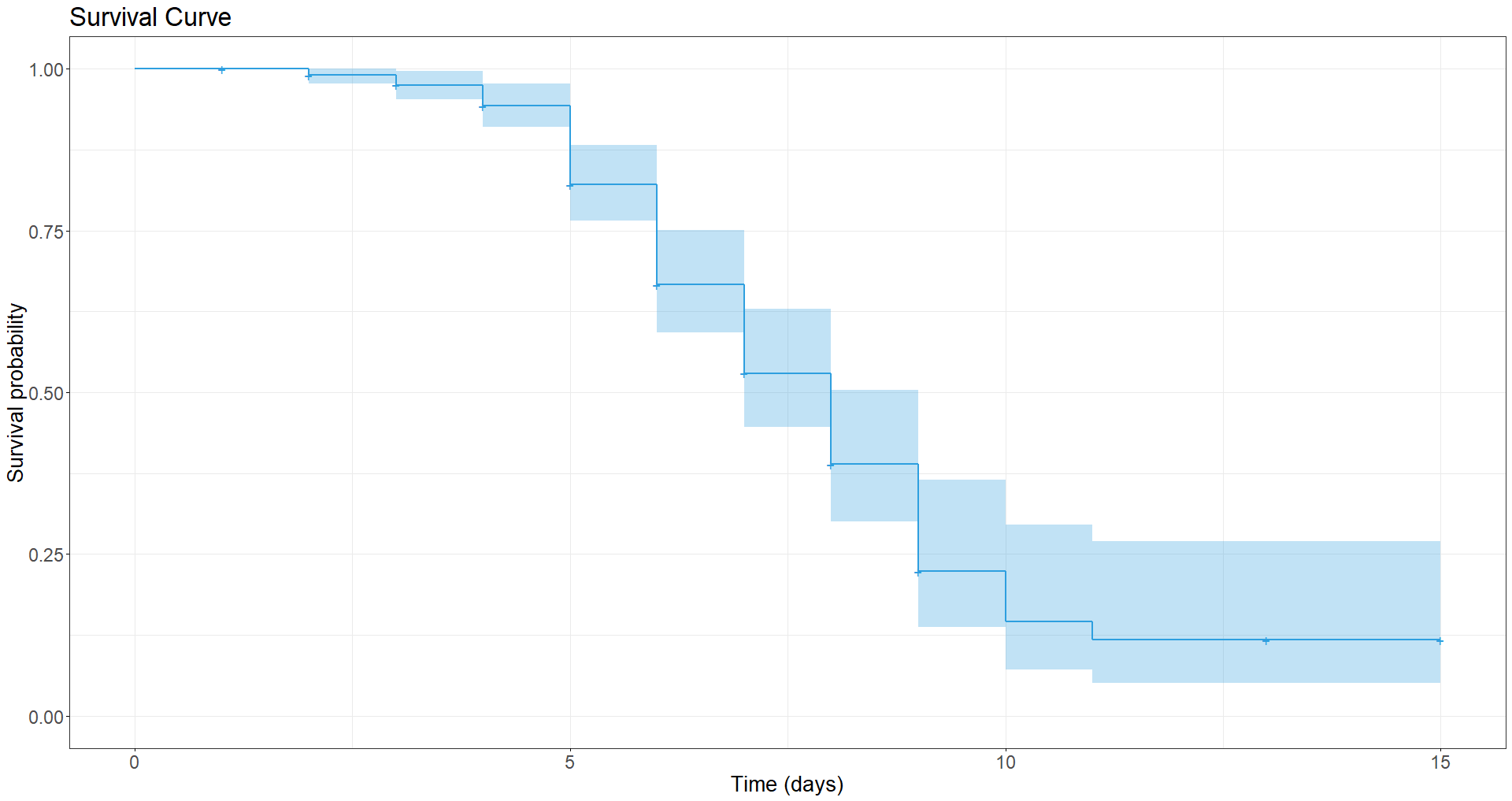

Supplement: Supplementary file 1 [file Table_1.DOCX]
